# Surgical Outcome of Spinal Neurilemmoma: Two Case Reports

**DOI:** 10.1097/MD.0000000000000490

**Published:** 2015-02-06

**Authors:** Kuang-Ting Yeh, Ru-Ping Lee, Tzai-Chiu Yu, Ing-Ho Chen, Cheng-Huan Peng, Kuan-Lin Liu, Jen-Hung Wang, Wen-Tien Wu

**Affiliations:** From the Department of Orthopedics (K-TY, T-CY, I-HC, C-HP, K-LL, W-TW); Department of Research (J-HW), Hualien Tzu Chi Hospital, Buddhist Tzu Chi Medical Foundation; Institute of Medical Sciences (K-TY, R-PL, W-TW); and School of Medicine (T-CY, I-HC, W-TW), Tzu Chi University, Hualien, Taiwan.

## Abstract

Neurilemmoma commonly occurs from the fourth to sixth decades of life with an incidence of 3 to 10 per 100,000 people, and is rare in adolescence. This case report describes the clinical and radiographic features of 2 rare cases with intraspinal neurilemmoma of the cervical and thoracic spine.

A 29-year-old man who experienced middle back pain with prominent right lower limb weakness, and an 11-year-old boy who suffered from sudden onset neck pain with left arm weakness and hand clawing for 2 weeks before admission to our department were included in this case report.

Magnetic resonance imaging of both patients revealed an intraspinal mass causing spinal cord compression at the cervical and thoracic spine. The patients subsequently received urgent posterior spinal cord decompression and tumor resection surgery. The histopathology reports revealed neurilemmoma. The 2 patients recovered and resumed their normal lives within 1 year.

Intraspinal neurilemmoma is rare but should be considered in the differential diagnosis of spinal cord compression. Advances in imaging techniques and surgical procedures have yielded substantially enhanced clinical outcomes in intraspinal neoplasm cases. Delicate preoperative study and surgical skill with rehabilitation and postoperative observation are critical.

## INTRODUCTION

Spinal intradural extramedullary tumors account for two thirds of intraspinal neoplasms and are primarily represented by meningioma and schwannoma.^1^ Neurilemmoma, or intraosseous schwannoma, typically occurs in the fourth to sixth decade of life and is a rare benign neoplasm that comprises <0.2% of primary bone tumors, with an incidence of 3 to 10 per 100,000 people.^2^ The most common axial skeleton site of involvement is the mandible, and rare cases involving the mobile spine have been reported.^3,4^ We report 2 cases of solitary intraspinal neurilemmoma that caused neurologic deficit, one at the cervical spine and the other at the thoracic spine.

## CASE REPORT

This study was approved by the Research Ethics Committee of Hualien Tzu Chi Hospital, Buddhist Tzu Chi Medical Foundation, Hualien, Taiwan. The informed consents of the 2 patients were included in the data for the ethics committee review.

### Case 1

**FIGURE 1:**
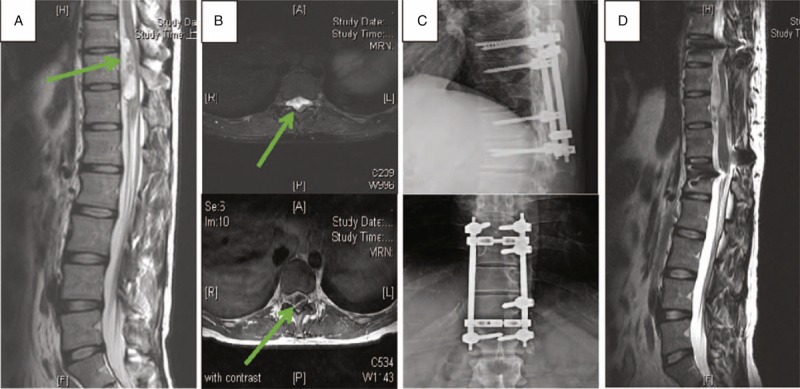
Radiological studies of Case 1. The green arrows mark the position of tumor. (A) Preoperative sagittal T2-weighted imaging of T-spine magnetic resonance imaging (MRI). (B) Preoperative axial imaging of T-spine MRI. Upper image is T2 weighted and lower image is Gd-enhanced T1 weighted. (C) Postoperative T-spine X-ray anteroposterior and lateral view. (D) Sagittal T2-weighted imaging of T-spine MRI at 2 y postsurgery.

A 29-year-old man, who is a telecommunications engineer, experienced back soreness and muscle cramping in the abdomen for 8 months. Progressive weakness of both lower limbs with an unstable gait was noted for 1 month before surgery. Numbness in the bilateral lower limbs and lower abdomen (below the umbilicus) occurred simultaneously. Positive myelopathy with prominent right lower limb weakness was noted at the outpatient department (OPD). The preoperative Nurick score was 3 and the visual analog scale (VAS) for upper back pain was 7. T2-weighted magnetic resonance imaging (MRI) of the entire spine revealed a 5.1 × 1.4 × 1.5 cm irregular mass from the vertebral body with a compressed spinal cord at the T10–T12 level. Irregular enhancement was observed in T1-weighted images after gadolinium administration (Figure 1A, B). Emergent surgical excision and decompression with T9-L1 posterior instrumented fusion was performed (Figure 1C). The tumor was observed to be well demarcated and extending into paravertebral areas and the spinal canal. No adhesions were identified between the dura and tumor without nerve involvement. A follow-up MRI at 3 years postsurgery revealed a patent spinal canal (Figure 1D).

### Case 2

**FIGURE 2:**
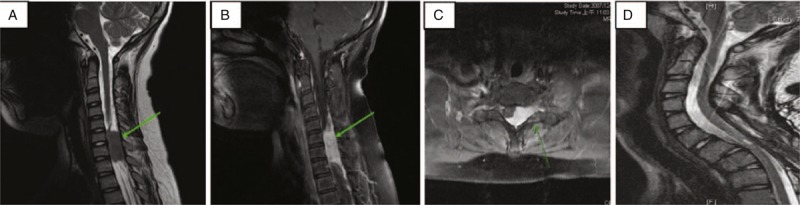
Radiological studies of Case 2. The green arrows mark the position of tumor. (A) Preoperative sagittal T2-weighted imaging of C-spine MRI. (B) Preoperative sagittal Gd-enhanced T1-weighted imaging of C-spine MRI. (C) Preoperative axial Gd-enhanced T1-weighted imaging of C-spine MRI. (D) Sagittal T2-weighted imaging of C-spine MRI at 3 y postsurgery.

An 11-year-old boy experienced the sudden onset of left forearm pain for 1 month after lifting heavy wood. Within 2 weeks, exacerbated neck pain, progressive left arm weakness, and a clawing hand were noted. His body leaned to the right side at that time. Left elbow flexion and extension weakness with ulnar side numbness was noted at the OPD. Positive spurling and abduction relief signs with knee and ankle jerk hyperreflexia were found. Acute myelopathy with radiculopathy was revealed. A T2-weighted MRI study revealed a 3.1 × 1.5 × 1.6 cm intraspinal mass at the level of C6–C7 with a compressed cervical cord on the left side (Figure 2A, B). Tumor enhancement was observed in T1-weighted images after administering gadolinium (Figure 2C). The preoperative Japanese Orthopedic Association (JOA) score and the Nurick score were 12 and 2, respectively. The VAS for neck pain was 8. We performed C5–C7 posterior tumor excision and decompression of the spinal cord by laminectomy. At the level of the sixth and seventh cervical vertebra, a well-bounded grayish white mass compressing the spinal cord was noted. The mass within the spinal canal was measured at approximately 1.2 cm in diameter. The tumor was not attached to any nerve root and was completely excised. A follow-up MRI at 5 years postsurgery revealed a patent spinal canal (Figure 2D). The time course of illness of the 2 patients is shown in Table 1.

**TABLE 1:**
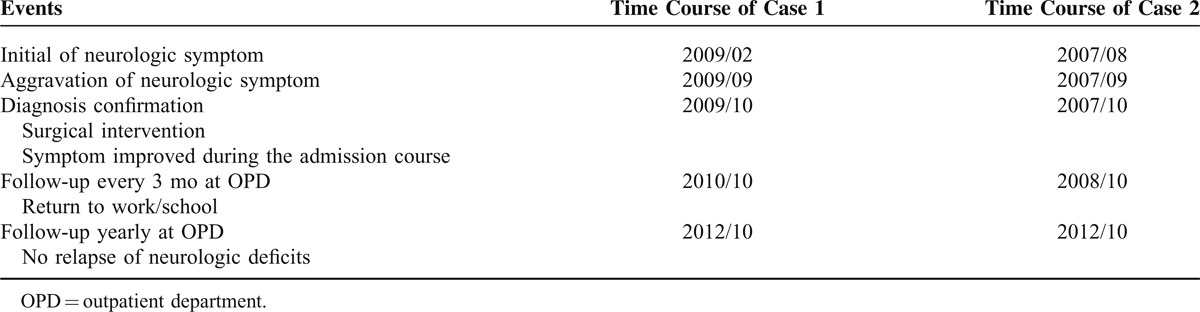
Timeline

## RESULTS

**FIGURE 3:**
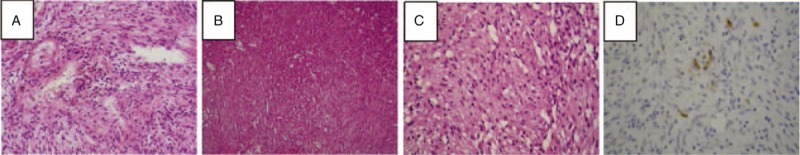
Histological features of Case 1 and Case 2. (A) Case 1: compact cellular area with spindle-shaped cells; palisading of nuclei represent Verocay bodies with areas of cellular myxoid connective tissue (hematoxylin and eosin [H&E] stain; ×100). (B) Case 2: compact cellular area with spindle-shaped cells (H&E stain; ×40). (C) Case 2: palisading of nuclei representing Verocay bodies with areas of less cellular myxoid connective tissue (H&E stain; ×100). (D) Case 2: immunohistochemistry stain indicating S100 (+).

In Case 1, the specimen was grayish and elastic. Microscopically, it exhibited neurilemmoma with Verocay body formation (Antoni type A tissue) accompanied by cystic, myxoid, and hemorrhagic degeneration (Antoni type B tissue) (Figure 3A). The man underwent operation and was discharged. The Nurick score improved from 3 to 0 and the VAS for back pain improved from 7 to 1 at 3 months postsurgery. Postoperative gait and sensation in the lower extremities exhibited excellent recovery.

In Case 2, the tumor tissue was gray and grossly elastic. Microscopically, it exhibited a well-defined spindle cell tumor with moderate-to-high cellularity (Antoni type A tissue) (Figure 3B). Focal staghorn vessels were also noted in the tumor area, and wavy nuclei were observed in certain tumor cells (Figure 3C). No increased mitotic figure was observed in the tumor. Immunohistochemistry studies revealed epithelial membrane antigen (−), CD34 (−), and S100 (focal +) (Figure 3D). Therefore, a cellular neurilemmoma was considered. The boy underwent surgery and the admission course was smooth. The JOA score and the Nurick score improved to 17 and 0, respectively, at 3 months postsurgery. The VAS for neck pain was 0. After a supportive rehabilitation program, he returned to school at 9 months postsurgery. Postoperative OPD cervical plain film follow-up revealed gradual onset of C5–C6 and C6–C7 spondylolisthesis, but his function remained favorable.

## DISCUSSION

Neurilemmomas are benign neoplasms that account for <0.2% of primary bone tumors.^5^ An osseous schwannoma affects the bone through 3 possible mechanisms: tumors arising within the nerve canal and forming a dumbbell-shaped configuration producing an enlarged canal, an extraosseous tumor resulting in bone erosion, or a tumor arising centrally within the bone.^6^ Sherman^7^ reported the presence of myelinated fibers within the marrow of vertebral bodies in 2 adults. Pederson et al^8^ noted nerve filaments of the recurrent branch of each spinal nerve root with the accompanying blood vessels entering the vertebral body. The most common site of occurrence is the mandible because of the long course of nerves within this bone.^9^ Reports of axial skeleton sites (eg, the vertebral body, rib, and sacrum) affected by neurilemmoma are rare.^10–12^

Spinal neurilemmoma often affects the spinal cord and causes radiculopathy or myelopathy symptoms. Detailed recording and thorough physical examination should be performed. Neglected and untreated myelopathy could cause irreversible neurologic deficit and disability. Computed tomography and MRI are the primary diagnostic tools for evaluating neurilemmomas.^13^ Gadolinium-contrast MRI images are generally suggested to help differentiate the neurilemmoma from fluid-filled cysts. Neurilemmoma is typically round or oval with a bright, heterogeneous signal on T2-weighted images, and a moderately bright signal on T1-weighted images. Technical advances in imaging techniques such as MRI and surgical procedures have substantially enhanced clinical results of intradural spinal tumor cases. For our 2 cases, we confirmed diagnosis of myelopathy on the basis of positive long tract sign and degrees of neurologic deficit. We arranged MRI with and without contrast to ensure the margin of tumor and the range of spinal cord compression, and then determined the surgical resection plans.

As with most benign neoplasms, neurilemmomas respond favorably to local resection surgery.^14^ Decompression and surgical resection of tumor are the primary therapy, with or without further fusion. For T-spine neurilemmoma of Case 1, we performed tumor resection with posterior instrumented fusion because the tumor was located at the physiologic kyphotic curve of the T spine, and postoperative progressive kyphosis could happen without instrumentation. For C-spine neurilemmoma of Case 2, we performed posterior decompression with tumor resection without instrumentation because of 2 reasons: the tumor was located at the physiologic lordotic curve and only involved the left side, and hence the right side facet joints could be preserved to maintain structure stability; and unnecessary additional fusion should be avoided to preserve the range of neck motion. The nerve is typically spread over the lesion, which is excised marginally to spare the nerve fibers. Interlesional resection is reasonable when complete resection might cause permanent loss of neurologic function. Most patients undergo resection surgery with complete relief of symptoms and recovery of neurologic functions. A low incidence of recurrence exists following complete resection, and malignant change is extremely rare in isolated lesions.^15^ After surgery, the removed tissues were pathologically examined and neurilemmoma was diagnosed. Both of our patients were satisfied with the functional recovery and resumed their normal lives well without residue neurologic deficit or local pain at regular annual follow-up.

## CONCLUSION

Spinal neurilemmoma that causes neurologic deficit is rarely reported. The recurrence of neurilemmoma is rare after careful surgical resection. In this article, 2 cases of spinal neurilemmoma with favorable middle-term follow-up functional outcome are reported. By careful physical examination, the level of neurocompression can be determined. MRI and surgical techniques enable resection. By pathologic examination, neurilemmoma can be diagnosed.
