# Surgical Outcome of Spinal Neurilemmoma: Two Case Reports: Erratum

**DOI:** 10.1097/01.md.0000464288.21217.a5

**Published:** 2015-04-10

In the article “Surgical Outcome of Spinal Neurilemmoma: Two Case Reports”,^1^ which appeared in Volume 94, Issue 5 of *Medicine*, Dr. Kuang-Ting Yeh's credentials were incorrectly listed as MD, PhD. Dr. Yeh is an MD and not a PhD. The article has since been corrected online.
